# A DNA methylation-based liquid biopsy for triple-negative breast cancer

**DOI:** 10.1038/s41698-021-00198-9

**Published:** 2021-06-16

**Authors:** Katrina Cristall, Francois-Clement Bidard, Jean-Yves Pierga, Michael J. Rauh, Tatiana Popova, Clara Sebbag, Olivier Lantz, Marc-Henri Stern, Christopher R. Mueller

**Affiliations:** 1 Queen’s Cancer Research Institute, Queen’s University, Kingston, ON Canada; 2 Department of Pathology and Molecular Medicine, Queen’s University, Kingston, ON Canada; 3 Circulating Tumor Biomarkers Laboratory, SiRIC, Translational Research Department, Institut Curie, Paris, France; 4 Department of Medical Oncology, Institut Curie, Paris, France; 5 Université Paris Descartes, Paris, France; 6 INSERM U830 Cancer, Heterogeneity, Instability and Plasticity (CHIP), Institut Curie, Paris, France; 7 INSERM CIC BT 1428, Institut Curie, Paris, France; 8 INSERM U932, Institut Curie, Paris, France; 9 Department of Biomedical and Molecular Sciences, Queen’s University, Kingston, ON Canada

**Keywords:** Breast cancer, Molecular medicine, Diagnostic markers, Predictive markers, Translational research

## Abstract

Here, we present a next-generation sequencing (NGS) methylation-based blood test called methylation DETEction of Circulating Tumour DNA (mDETECT) designed for the optimal detection and monitoring of metastatic triple-negative breast cancer (TNBC). Based on a highly multiplexed targeted sequencing approach, this assay incorporates features that offer superior performance and included 53 amplicons from 47 regions. Analysis of a previously characterised cohort of women with metastatic TNBC with limited quantities of plasma (<2 ml) produced an AUC of 0.92 for detection of a tumour with a sensitivity of 76% for a specificity of 100%. mDETECT_TNBC_ was quantitative and showed superior performance to an NGS *TP53* mutation-based test carried out on the same patients and to the conventional CA15-3 biomarker. mDETECT also functioned well in serum samples from metastatic TNBC patients where it produced an AUC of 0.97 for detection of a tumour with a sensitivity of 93% for a specificity of 100%. An assay for *BRCA1* promoter methylation was also incorporated into the mDETECT assay and functioned well but its clinical significance is currently unclear. Clonal Hematopoiesis of Indeterminate Potential was investigated as a source of background in control subjects but was not seen to be significant, though a link to adiposity may be relevant. The mDETECT_TNBC_ assay is a liquid biopsy able to quantitatively detect all TNBC cancers and has the potential to improve the management of patients with this disease.

## Introduction

The detection of circulating tumour DNA (ctDNA) has the promise to be a useful tool that is able to help guide cancer patient management^1–3^. Dying cancer cells^4^ release ctDNA into the blood carrying with it the same molecular markers as in the tumour^5,6^. These analyses have been found to have utility in early detection^7^, monitoring response to treatment and determining prognosis^1,5,8,9^. These assays have becoming increasingly complex and to date have primarily been focused on the identification of tumour-specific mutations^10^. Significant technical issues have, and will continue to, limit the overall sensitivity and specificity of mutation-based approaches. These include elevated background levels due to the presence of high concentrations of normal DNA^11^, high error rates in both PCR and sequencing-based approaches^8,12^ and limited sensitivity due to the small number and low frequency of available mutations^13–15^. A variety of approaches have been used in an attempt to mitigate these issues including BEAMing^16,17^ and sequencing error suppression assays^18^, but fundamental issues continue to limit the efficacy of these tests^19^.

DNA methylation is proving to offer significant advantages over mutation detection for the analysis of ctDNA^20–22^. It has long been known that during the process of tumourigenesis gene-specific hypermethylation and genome-wide hypomethylation occurs^23^. Hypermethylation is thought to be associated with the inactivation of tumour suppressor genes, which provide the tumour with growth advantages^23^. These hypermethylation events may occur relatively early in the transformation process^23,24^ and are generally frequent and consistent within tumour types^25–28^. Regions of hypermethylation have proven to be useful biomarkers for ctDNA in a variety of cancer types as these events are both numerous and consistent across a particular tumour type^29^. Methylation-based assays are being developed in many cancer types^30^ and a number of pan-cancer tests are also showing promise^31,32^.

The ultimate sensitivity of any liquid biopsy is determined by the total number of regions consistently detected by that assay. This is because, at a ctDNA concentration below one genome equivalent per volume of blood, individual targets of the assay may not be present in the sample^33^. To produce a test with optimal detection abilities we have identified large numbers of highly frequent tumour-specific methylation events that occur over a small region of the genome and used them to create a highly multiplexed next-generation sequencing (NGS)-based assay for the detection of tumour-specific hypermethylation in blood, which we have named methylation DETEction of Circulating Tumour DNA (mDETECT). Features of this test include a rigorous selection process, which identified tumour-specific methylated target regions of high CpG density that are present in at least 50% of tumour subtypes and that were not methylated in multiple normal tissues or in multiple blood cell subtypes. It incorporates a methylation biased PCR amplification process that enhances methylation signals and the sequencing of multiple CpGs in each probe ensures specificity in the presence of large amounts of normal DNA and, when combined with the large number of probes allows for extremely low levels of detection. Each mDETECT assay is specific to a particular type of cancer and using triple-negative breast cancer (TNBC) as a model we demonstrate that this test is superior to a *TP53* mutation-based assay in the metastatic setting and functions well using both plasma and serum.

## Results

### Identification and characterisation of tumour-specific hypermethylated regions

**Fig. 1:**
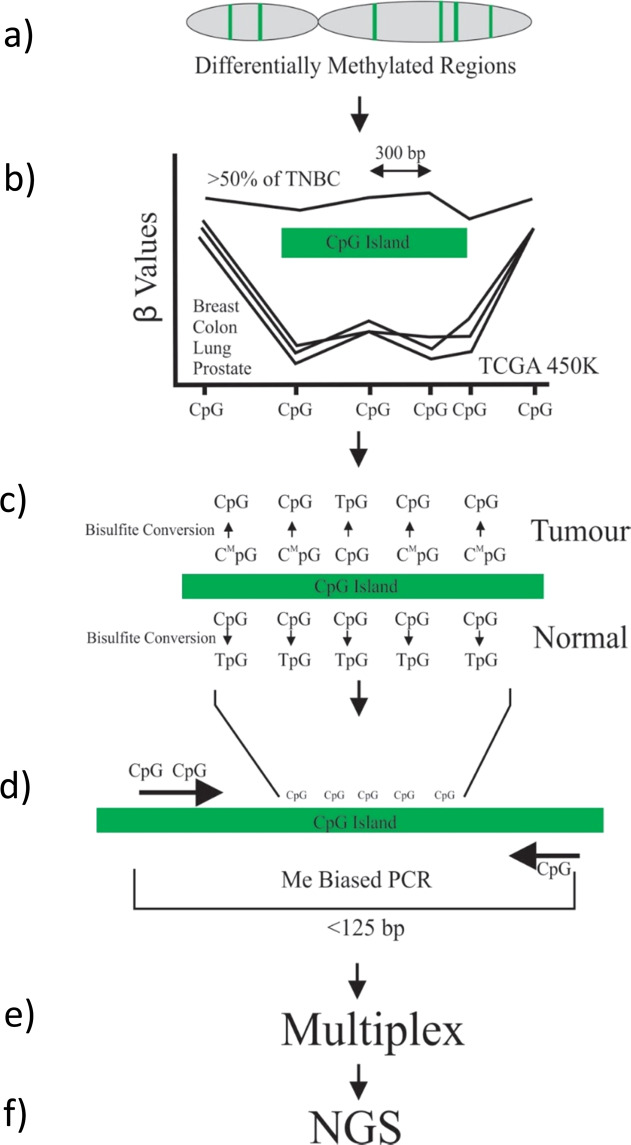
Overview of the mDETECT assay. **a** mDETECT relies on the blood-based detection of large numbers of differentially methylated regions that are specific to tumours represented by the green stripes on the grey chromosome. **b** To identify these regions with a high density of tumour-specific methylation, those CpG dinucleotides found to show high levels of methylation in the tumour populations but low levels in the normal tissues were identified as being differentially hypermethylated. β-values represent methylation levels at a given probe (CpG dinucleotide). **c** Bisulfite conversion of the DNA samples of these differentially methylated regions allows the multiple CpG residues in each region to be assessed and subsequently allows for differentiation between tumour and normal samples. **d** Methylation biased primers, as indicated by the arrows, are designed to preferentially but not exclusively amplify methylated DNA. This ensures that small amounts of methylated tumour DNA are amplified even in a high background of normal DNA. These PCR “probes” are designed to amplify products less than 125 bp (<125 bp) due to the highly fragmented nature of ctDNA (**e**). PCR is carried out by various types of multiplexing. **f** Next-generation sequencing (NGS) is carried out to identify multiple CpG dinucleotides that were methylated and to allow for high-level multiplexing.

**Fig. 2:**
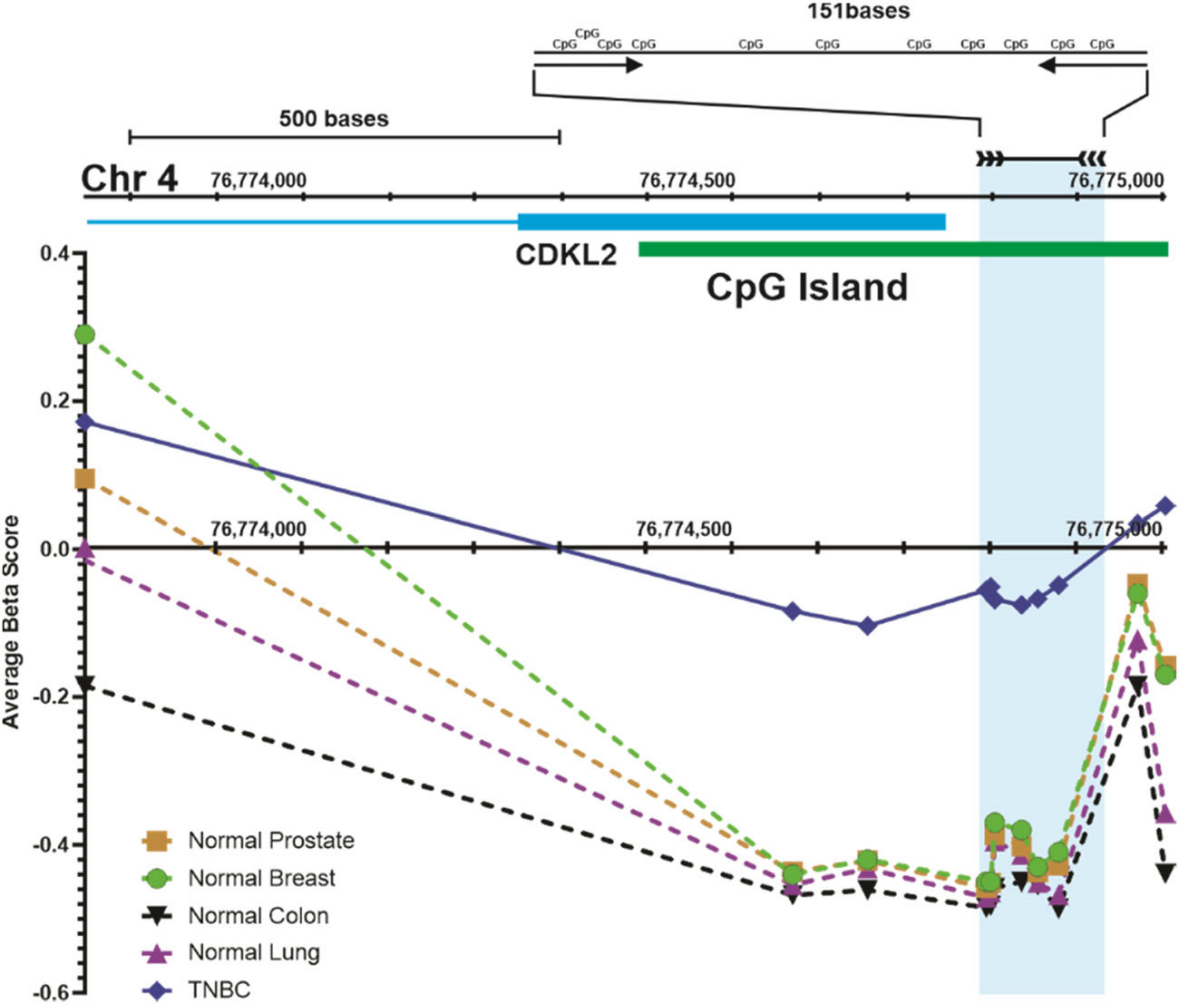
Methylation in the promoter region of the gene *CDKL2* in tumour and normal samples. Identification of differentially methylated target regions. Illumina 450 K methylarray probe beta values from various TCGA databases in the *CDKL2* promoter were averaged (average beta value) for normal prostate (normal prostate), colon (normal colon), lung (normal lung), and breast (normal breast) tissues, and for triple- negative breast cancer (TNBC) from selected TCGA datasets. The methylation values for the each of these samples were plotted relative to genomic position on the *x*-axis (hg18 nucleotide position on Chromosome 4) to determine the extent of differential methylation in surrounding areas. The first exon of the CDKL2 gene is shown (blue box) along with the identified CpG island (CpG Island in green). The location of the *CDKL2* probe is shown (light-blue box) and the expanded region shows the location of CpG residues in relation to the PCR primers (arrows) and product.

TNBC is an aggressive form of breast cancer with few targeted therapies and generally poor outcome^34^ and is an ideal target for the development of an mDETECT assay. It is also sufficiently different from other subtypes of breast cancer at the methylation level to require its own assay, likely due to its origin from basal cells^35^. Illumina 450k methylation data from the TCGA database was selected for tumours of the TNBC subtype based on both the Basal PAM50 molecular signature as well as ER, PR and HER2-negative histological status and data from tumours with *BRCA1* mutations were also analysed. Tumour-specific hypermethylated regions with a high density of CpG residues in a small area were identified based on at least 2 methylated CpG residues in the TCGA dataset being within 300 bp of each other and where there was tumour-specific hypermethylation in the majority of samples (>50%) but no methylation when compared to normal samples from breast, prostate, colon and lung tissues (Fig. 1a, b) (see “Methods” for details). The lack of methylation of these regions in a variety of blood cells was also assessed using published bisulphite whole-genome sequencing data^36^. These regions were predominantly associated with CpG islands, typically occurring around the CpG island shore and generally had a high CpG density. An example is shown for the *CDKL2* gene (Fig. 2). A total of 64 hypermethylated regions were identified and 6 additional regions were added that have been observed to be prognostic for TNBC^27,37^, as well as the *BRCA1* promoter region, which has implications for TNBC treatment^38,39^ and prognosis^40^. Optimal PCR primers were designed based on the bisulphite converted sequence where all CpG residues were considered to be methylated and typically results in several CpG residues in each primer though not generally at the 3’ position. This results in these primers having a bias towards amplifying methylated sequences but some are still able to amplify unmethylated sequences (Fig. 1d). These primers were designed to amplify regions less than 150 bp due to ctDNA being highly fragmented^41^.

**Fig. 3:**
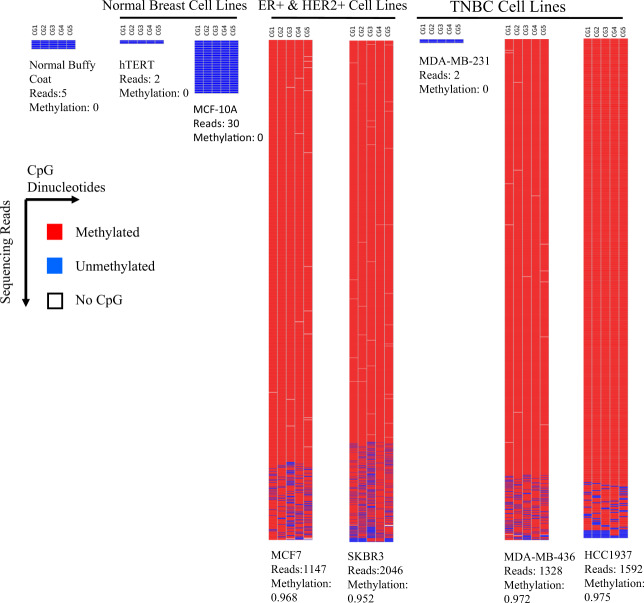
Singleplex sequencing results for the probe NFIC-A in cell lines. Shown here are the BiQ Analyser generated heatmaps of the NGS reads for the probe NFIC-A as analysed in numerous cell lines. Each column represents one of the 5 CpG dinucleotide in the amplified region while each row indicates an individual sequencing read. Blue fill indicates that the CpG dinucleotide was not methylated in a particular read while red fill indicates that the CpG dinucleotide was methylated in a particular read. The number of reads and the fraction that are methylated in each probe is indicated below each map. As expected, there is low methylation in the immortalised mammary epithelial cell lines 184-hTERT and MCF-10A, as well as the normal buffy coat DNA. Two of three TNBC cell lines (MDA-MB-231, MDA-MB-436, and HCC1937) were methylated demonstrating heterogeneity between individual cancers. Furthermore, MCF7 and SKBR3, an ER + and HER2 + cell line show methylation, indicating that the probe is not exclusively methylated in TNBC but also in other subtypes of cancer. In general, methylation was an all or none phenomena.

The primers were assessed using individual PCR amplification reactions (Singleplex PCR) using multiple breast cell lines and in normal buffy coat DNA (which is the source of most circulating DNA), followed by NGS sequencing. Only CpG residues between the primers were assessed and contained between 3 and 21 CpG residues ensuring the detection of genuinely methylated regions. An example of the Singleplex analysis for the *NFIC* promoter region (with five CpG residues) is shown in Fig. 3. The majority of reads were found to either be entirely methylated or entirely unmethylated at all of the CpGs in the sequenced region. This analysis resulted in the development of a total of 86 probes corresponding to 71 regions.

### mDETECT_TNBC_ characterisation of TNBC tumours

**Fig. 4:**
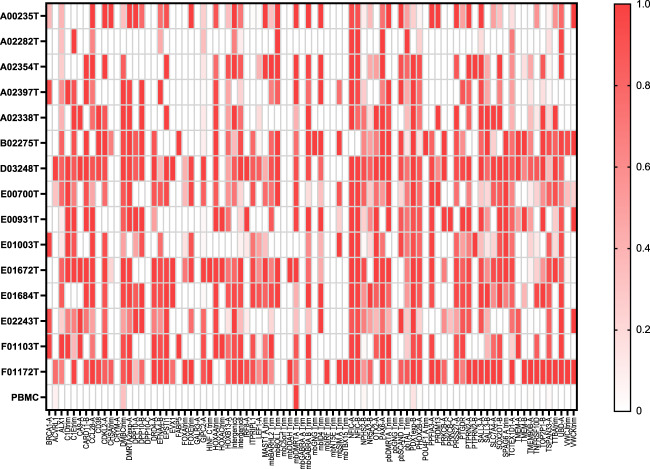
mDETECT analysis of 15 TNBC tumours. The shading indicates the fraction of reads exhibiting methylation at all CpG residues in the region between primers compared to all reads, for the 86 probes in each of the 15 TNBC tumours analysed, as well as PBMC DNA from a normal individual. Each column represents an individual probe while each row represents a different tumour sample. Between 18 and 65 probes had a methylation fraction above 0.1 for each sample (average 46) while the PBMC sample had 2 positive probes.

The probes remaining after optimisation were then multiplexed together and assayed on a collection of 15 primary TNBC tumours from the Ontario Tumour Bank (OTB). The BiQ Analyser^42^ program was used to derive methylation levels and patterns for each of the probes. BiQ Analyser filters individual reads and was set to pass only those that had a 90% homology to the reference sequence and where all CpGs between the primers were identifiable. Reads were then counted that were methylated at all CpGs in that read and the fraction of these reads verses all reads was calculated and is shown as a heatmap for each probe and sample (Fig. 4). The distribution of this methylated fraction for all of the probes indicated that a fraction of 0.1 divides positive from negative probes when compared to normal PBMC DNA (Supplementary Fig. 1). Based on this cutoff between 18 and 65 of the 86 probes per patient were positive for methylation (average 46) but only 2 were positive for normal PBMC DNA (Fig. 4). This demonstrates that the mDETECT_TNBC_ assay is able to recognise a wide range of TNBC tumours with a substantial number of probes reporting for each one. Based on these results the number of optimally preforming probes was reduced to 68.

### Multi-Singleplex version of the mDETECT_TNBC_ assay

**Fig. 5:**
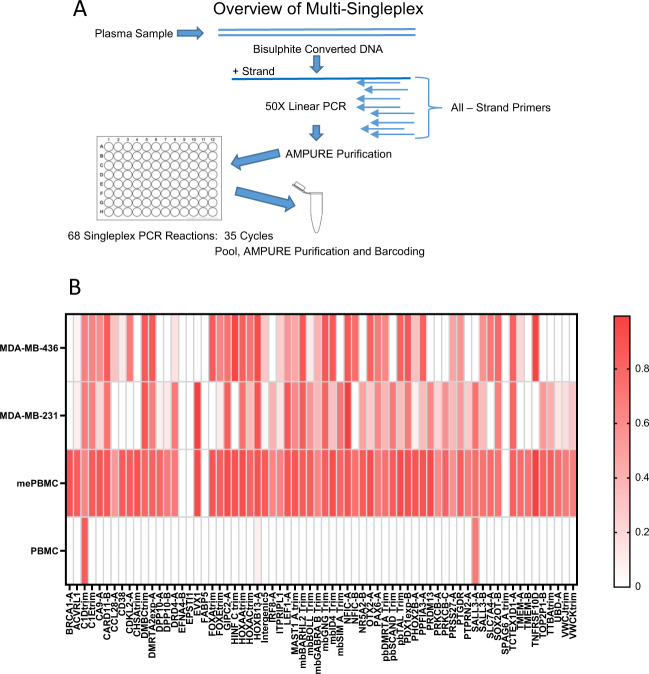
Multi-Singleplex approach. **A** Samples were linear amplified using all 68 negative strand primers for 50 cycles. After purification the sample was split into 68 separate PCR reactions with each probe. The products were then pooled and barcoded. **B** A heatmap of the fraction methylated DNA for each probe. Two TNBC cell lines (MDA-MB-436 and 231), normal PBMC DNA and PBMC synthetically methylated (mePBMC) were analysed. For each probe the number of reads methylated at all CpGs positions between the primers was divided by the total number of reads to give the fraction of methylation for each with the shading representing this fraction from 0 to 1.

The multiplex protocol used above generated considerable inequity in the number of reads for each probe, as well as high levels of primer dimer. To address this issue, we carried out individual PCR reactions for each probe set. To avoid dilution of the sample we preformed 50 rounds of linear PCR to produce sufficient copies of the original molecules to be divided amongst the 68 PCR reactions (Fig. 5A). Individual PCR reactions were then pooled and barcoded together. We refer to this technique as Multi-Singleplex and it produced a more even distribution of reads between the different probes, essentially eliminated primer-dimers, and because of the reduced competition between different primers results in the presence of both methylated and non-methylated reads for most probes, an advantage in confirming the functionality of the test. Once these regions are validated it is expected that the use of an approach such as RNase H-dependent PCR (rhPCR) will allow for the reduction of this test to a single tube assay^43^. PBMC DNA as well as synthetically methylated DNA (mePBMC) and the MDA-MB-231 and MDA-MB-436 TNBC cell lines were analysed with an average sequencing depth of 630,000 reads. An average of 82% of these reads passed the BiQ Analyser criteria confirming the high quality of the products. In the PBMC sample, probes with very low read numbers (1–10 reads) appear to be methylated (Supplementary Fig. 2) but this is not observed when read numbers are higher for a given probe and was not observed in the mePBMC or either of the TNBC cell line samples. These products may be from partially bisulfite converted DNA that are seen when there is no competition from other DNA or are diluted out by legitimate products. This was not observed if there were more than 100 reads for an individual probe, so in a given sample those probes with <100 reads where not considered. Based on this and the positive threshold of the methylated fraction being at or above 0.1 some 47 and 43 of the probes (out of 68) were positive for the TNBC cell lines while only two probes were positive for normal PBMC DNA.

### mDETECT_TNBC_ characterisation of TNBC patient plasma samples

**Fig. 6:**
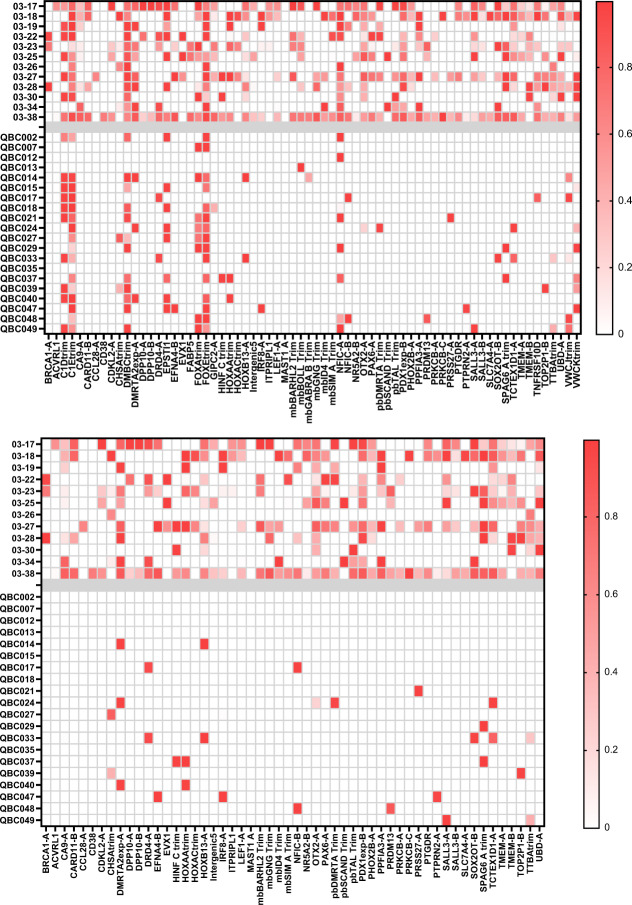
Test cohort. Twelve plasma samples from Cohort 03 patients and 20 plasma samples from healthy age-matched volunteers (QBC 002-049) were analysed for mDETECT as described in Fig. 5 and a heatmap of the fraction of methylated DNA for each probe is shown. (Top) The original 68 probes set is shown. (Bottem). Probes were removed based on the presence of 3 or more positive probes in the controls to leave 54 probes.

A previously characterised cohort of metastatic TNBC patients^44^ (Cohort 03) was used to assess mDETECT_TNBC_ using plasma samples from these patients and plasma from a cohort of age-matched healthy women (Queen’s Breast Control (QBC)). A selection of 12 patient samples and 20 age-matched controls were used as a test cohort to assess the performance of individual probes (Fig. 6A). The average sequencing depth for the Cohort 03 samples was 850 k reads with a yield of high-quality reads of 55% while the QBC samples had an average depth of 701 k reads with a 61% yield. A number of probes demonstrated significant background in multiple healthy control, for example, both C1D and C1E probes as well as both FOXA and FOXE probes. Probes with three or more positive normal samples as well as those with poor technical performance were eliminated to give a core of 54 probes, including *BRCA1* (Fig. 6B) (Supplementary Table 1). This finalised assay covers 6500 bp of the genome and interrogates 433 CpG residues between the PCR primers. The primers on average have 2.4 CpGs in them, accounting for the methylation bias. Based on TCGA data from primary breast cancer, each TNBC patient is on average positive for 31 probes, and most probes are also positive in a high fraction (average 57%) of other breast cancer subtypes (Supplementary Table 1). For the Cohort 03 samples on average 19.25 probes were positive but only 1.5 for the QBC controls. Considering the number of positive probes as a combined Methylation Index this gave the test an AUC of 0.97 with 92% sensitivity and 100% specificity (Supplementary Fig. 4). *BRCA1* methylation was not considered in this or subsequent AUC analyses.

**Fig. 7:**
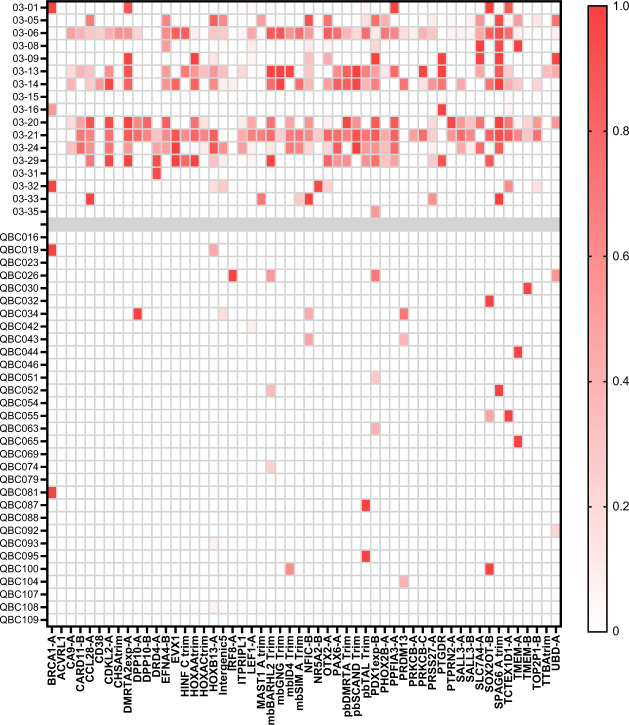
mDETECT analysis of Validation Cohort 03 samples and new control plasma DNA. The DNA from 17 patients not included in the Test Cohort, as well as 31 new plasma samples from healthy age-matched volunteers (QBC 16-109) were analysed using mDETECT for the 54 optimal probes as described in Fig. 6. A heatmap of the fraction of methylated DNA for each probe is shown as described in Fig. 5.

As a validation cohort 17 samples from patients not in the test series of the Cohort 03 samples as well as 31 independent age-matched QBC controls were assayed, however, the available material was extremely limited and included plasma that had only been spun at low speed as well as much smaller aliquots of plasma, as little as 0.5 ml (Supplementary Fig. 3). The average sequencing depth for the Cohort 03 samples was 1.7 million reads with 53% of reads passing the BiQ Analyser filter and the QBC samples had an average depth of 1.5 million reads with a 49% yield of high-quality reads. There was an average of 14.5 positive probes for Cohort 03 verses 0.9 positive for the QBC controls (Fig. 7). Despite the limitations of these validation samples the mDETECT_TNBC_ assay produced an AUC of 0.92 with a sensitivity of 76% and a specificity of 100% (Supplementary Fig. 4).

**Fig. 8:**
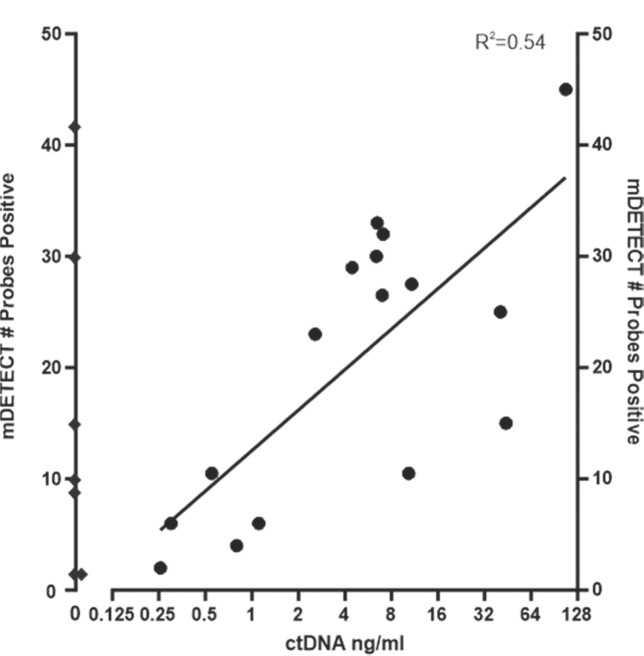
mDETECT versus ctDNA levels determined by TP53 mutation sequencing. A next-generation sequencing TP53 mutation-based test was previously used to quantitate ctDNA levels in the plasma from these patients and is expressed on a log scale as ng of ctDNA per ml of plasma (*x*-axis). The number of mDETECT probes that were positive for methylation for each sample is plotted (right *y*- axis). For samples that were negative for the TP53 assay (Diamonds) the mDETECT levels are plotted on the left *y*-axis as zero cannot be plotted on a log scale. Regression analysis shows that the relationship between mDETECT and TP53-based levels is reasonably well correlated (*R*^2^ = 0.54).

Cohort 03 had previously been characterised for ctDNA levels using a TP53 next-generation sequencing assay^44^. Of seven samples that had identifiable TP53 mutations but were still negative based on sequencing, all were positive for mDETECT_TNBC_ (Fig. 8, left axis) and 5 of these likely had some ctDNA present as the level of CTCs in these patients was from 2 to 5580 (per 10 ml). The mDETECT_TNBC_ levels were correlated with ctDNA levels measured by TP53 mutant allele frequency (where observed) with an *R*^2^ value of 0.54 (Fig. 8). Replicate samples were in good agreement and on average were within 30% of each other (Supplementary Fig. 3).

### mDETECT_TNBC_ functions well in serum samples

**Fig. 9:**
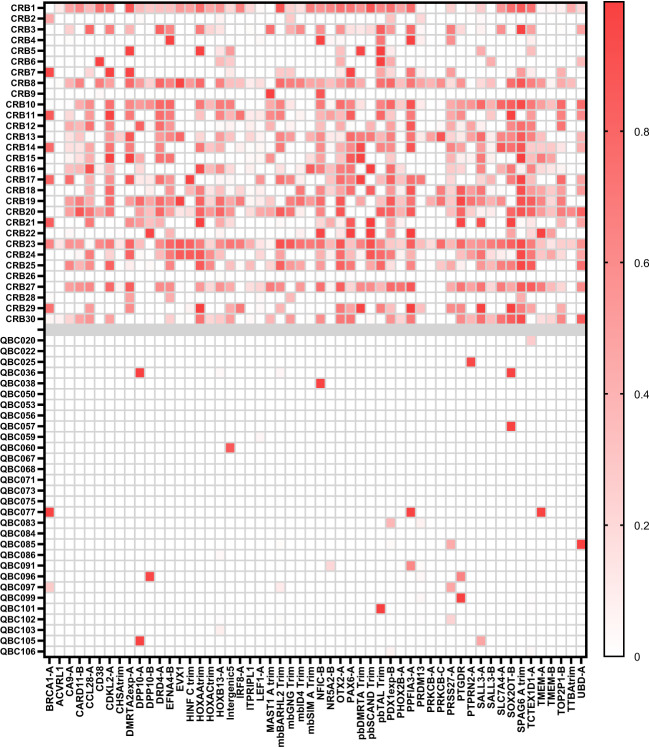
Analysis of serum samples from metastatic TNBC patients. Thirty serum samples from metastatic TNBC patients (CRB1–30) and 30 new serum samples from healthy age-matched volunteers (QBC020–106) were analysed using mDETECT for the 54 optimal probes as described in Fig. 6. A heatmap of the fraction of methylated DNA for each probe is shown as described in Fig. 5.

Most liquid biopsy assays have used plasma in an effort to reduce background from normal cell free DNA (cfDNA) that limits their sensitivity. The mDETECT_TNBC_ assay is resistant to contaminating DNA due to the preferential amplification of methylated ctDNA. To demonstrate this, we further validated the assay in an independent cohort of 30 metastatic TNBC patient serum samples (CRB) along with 30 new serum QBC control samples (Fig. 9). The average sequencing depth for the CRB samples was 1.1 million reads with 75% of reads passing the BiQ Analyser filter and the QBC samples had an average depth of 1.8 million reads with a 69% yield of high-quality reads. On average the CRB samples had 23.7 positive probes (range 0 to 50) while the QBC had only 0.77 positive probes (range 0 to 2). The assay produced an AUC of 0.97 and at a threshold of 2.5 positive probes achieved a sensitivity of 93% for a specificity of 100% (Supplementary Fig. 4), which represented 2 of the 30 patient samples being below this threshold. These patients, CRB 9 and 26 (mDETECT_TNBC_ of 2 or 0, respectively), had normal CA15.3 levels (18 or 21), normal CEA (2 and 2) and were the only patients to have had no previous lines of therapy.

### BRCA1 promoter methylation

While *BRCA1* mutations are associated with the TNBC subtype, methylation of the *BRCA1* promoter is more frequent than mutations and has similar predictive and prognostic value^45^. For those reasons this region was included in the mDETECT_TNBC_ assay but was not part of the probe assessment for mDETECT_TNBC_ as it did not meet the 50% frequency criteria for inclusion. Seven out of 28 Cohort 03 samples, and 8 out of 30 CRB samples, were positive for BRCA1 methylation or ~25%, which is in keeping with reported levels in the TNBC patient population^46^. Complicating the interpretation of this observation is that 4 out of 81 (5%) control samples were also positive for *BRCA1* methylation. These women had an increased risk of a previous abnormal mammogram (Fisher Exact test *p* = 0.095) and also had a significant risk of having had a previous breast biopsy (Fisher Exact test *p* = 0.047). Of these women with suspicious findings one had an mDETECT_TNBC_ score of 2 while 2 had a score of 3, placing them as borderline positive. This suggests that *BRCA1* methylation status can be determined by mDETECT_TNBC_ but only in the context of a positive finding by the rest of the panel, but its link to a pre-malignant or benign disease is currently unclear.

### Background methylation in the control population

The sensitivity of any assay is dependent on the background levels in normal populations so we wanted to investigate further the sources of potential background. The positive probes seen in the QBC controls seem to be authentic in that they have both high read numbers and a high fraction of methylated DNA, but analysis of all 81 samples suggests its occurrence is random in nature with no association between methylation of different probes, even those derived from the same region of the genome (Supplementary Fig. 5). Some 20 of the 54 probes are not methylated in any control sample and 58 of 81 (72%) of the control samples have only 0 or 1 positive probe each. On average each probe is methylated in 1.5 women. Eliminating probes with higher background would improve the test performance but with the potential loss of sensitivity. Given these were aged matched normal samples (average age 58 years) it is possible that some of these women with higher mDETECT_TNBC_ scores could have undiagnosed breast cancer. We also speculated that this background could be due to Clonal Hematopoiesis of Indeterminate Potential (CHIP), which is both common in an aging population^47^ and is associated with abnormal global methylation akin to a CpG Island Methylator Phenotype (CIMP)^48^. We analysed PBMC DNA from six QBC controls with high mDETECT_TNBC_ levels (2 to five positives, avg 3.2) for a panel of CHIP related mutations. One of these women (with three positive probes) had a characteristic TET2 c.679 G > T (p.Glu227Ter) variant at an allele fraction of 4.27%, indicating she had CHIP. However, of the three positive probes, two were found in two different women and the third was unique to her. This suggests that CHIP does not result in a generalised background and will likely not be a major issue for this type of methylation-based assays. Interestingly, there was a correlation between the number of positive mDETECT_TNBC_ probes and weight and BMI in this population (Pearson correlation 0.296, *p* = 0.008, and 0.270 *p* = 0.015), suggesting a link to adiposity and raises the possibility that a weight derived threshold for the test could be defined.

### Correlation with CA15-3

**Fig. 10:**
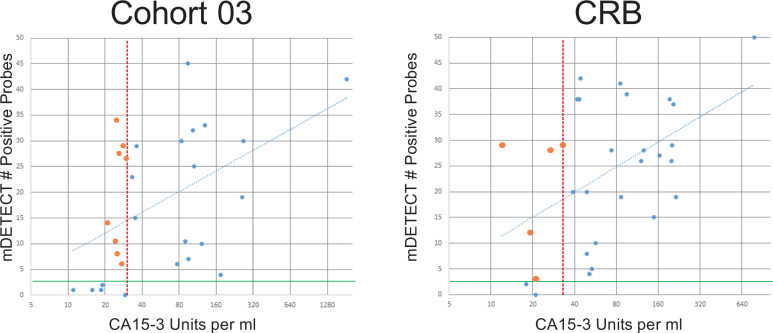
Comparison of mDETECT and CA15-3 detection. The levels of mDETECT and CA15-3 were compared for patients in both Cohort 03 and CRB. The normal range of CA15-3 is up to 30 units per ml (red-dashed line) while the threshold for mDETECT is 2.5 positive probes (Solid green line). In the Cohort 03, eight patients that were negative for CA15-3 were positive as determined by mDETECT (orange circles). Similarly, five patients in the CRB cohort that were negative by CA15-3 were positive by mDETECT (orange circles). The mDETECT and CA15-3 levels were somewhat correlated with an *R*^2^-value of 0.225 for Cohort 03 and 0.238 for the CRB cohort (blue dashed lines).

CA15-3 is a commonly used marker for following tumour burdens in metastatic breast cancer patients but is know to preform poorly^49,50^. In Cohort 03, 8 women (out of 29, 28%) who were negative for CA15-3 (<30 units/ml) were positive for mDETECT, and similarly in the CRB cohort 5 women (out of 30, 17%) who were negative for CA15-3 were positive for mDETECT (Fig. 10). A number of these patients who were CA15-3 negative had high levels of ctDNA as measured by both mDETECT and TP53 mutation assays. mDETECT is somewhat correlated with CA15-3 with *R*^2^ values of 0.225 and 0.238 for the respective cohorts. Levels of mDETECT were not correlated with any other clinical variable.

## Discussion

The mDETECT system has the potential to be targeted to any cancer subtype to ensure that the test is as specific and sensitive as possible. This is particularly important for malignancies such as breast cancer that are known to include numerous subtypes likely to have distinct methylation patterns^28^. Our analysis of TCGA data indicated that a pan-breast cancer test would have dramatically under detected TNBC and is in keeping with basal breast cancers, which largely overlap with TNBC, having a different aetiology^35^. Analysis of the individual regions making up the mDETECT_TNBC_ in the TCGA dataset indicates that on average Basal cancers were positive for 53% of the probes (87 samples), with no tumour being positive for <11 probes and on average each was positive for 30.5 probes (Supplementary Table 1). Interestingly, the TNBC assay does recognise the other breast subtypes with high frequency with 66% positive for Her2 tumours (31 samples), 46% positive for Luminal A tumours (288 samples) and 63% positive for Luminal B tumours (127 samples) (Supplementary Table 1). By developing a large number of well characterised targets that are frequently methylated in TNBC; using high levels of multiplexing achieved by targeted PCR combined with NGS; detecting multiple CpG methylation events in a given read to circumvent the error rates associated with NGS, mDETECT allows for sub-genome levels of ctDNA detection, which is required for detection of small tumours^33^.

The TNBC version of mDETECT_TNBC_ incorporates a BRCA1 methylation probe due to the predictive and prognostic value of this frequent event in TNBC, which is associated with a better outcome^46,51^ and may be correlated with sensitivity to olaparib^52,53^. Our observation of a significant level of BRCA1 methylation in healthy controls is consistent with its previous observation in the white blood cells of healthy women^54,55^, suggesting the origin of this signal may be from hematopoietic cells rather than ctDNA. Whether this is an inherited epimutation that represents a risk factor for the development of breast cancer^56,57^, or is a spontaneous event that may represent a “first hit”^58^, has not been resolved^57^. Its relatively weak association in our control population with an abnormal mammogram and previous breast biopsy indicates further study will be needed to determine its role as a marker of a benign or pre-cancerous state. Clearly its use as a blood-based marker must be correlated with the presence of a TNBC signature.

The origin of the sporadic occurrence of methylation seen in the healthy controls is not clear. Increased methylation levels, typically measured in the blood, have been associated with aging and risk of death^59^ and there is some evidence for it being linked to increased cancer-related death^60^. Several studies have suggested that accelerated epigenetic aging may be linked to increased risk of breast cancer^61,62^. We did not observe a correlation between mDETECT_TNBC_ signals and age in our control population but did see a significant association with weight and BMI, which is interesting when considering the possible interplay between epigenetic changes, obesity and breast cancer risk^63^. An increased risk of developing CHIP is also associated with aging^47^ and it is thought to be an epigenetic disorder with altered DNA methylation^48^, and due to its prevalence was considered to be a possible source of background. While one of six women with slightly increased background methylation was observed to have CHIP the methylation pattern in this patient was not present in the rest of the control cohort, suggesting CHIP is not a significant factor or does not produce a consistent pattern of methylation. The current design of mDETECT_TNBC_ tends to produce an “all or none” signal for each probe, so it is possible that this background methylation may represent much less starting material than the signal seen in ctDNA. Incorporation of a Unique Molecular Identifier (UMI) into this system should resolve this issue and may improve the test’s performance.

Compared to mutation-based assays, DNA methylation offers more frequent and consistent targets that are able to offer better overall test performance. While methylation assays based on single regions have limited functionality, the use of multiple targets has been shown to increase detection^64^. A ten gene panel assessed by a QM-MSP method called cMethDNA achieved a sensitivity of 91% with a specificity of 96% (area under the curve; AUC = 0.994; *p* < 0.0001) in recurrent metastatic breast cancers^65^. A six-gene sub-panel of cMethDNA was able to predict survival in women with metastatic disease (PFS 1.79 *p* = 0.002, OS 1.75, *p* = 0.003)^66^. However, MSP like assays have restricted performance as reflected in the elimination of 4 regions in the cMethDNA assay due to a high coefficient of variation^66^ and this assay failed to predict pathological response based on serum^67^. A method using the targeted sequencing of three regions uniquely methylated in normal breast tissue achieved an AUC of 0.9125 with a sensitivity of 80% and a specificity of 97% in localised cancer and was able to monitor treatment response^68^.

Methylation-based pan-cancer approaches have included the use of methylated DNA immunoprecipitation (cfMeDIP-seq) followed by whole-genome sequencing, which yielded high discriminatory power in multiple cancers, though breast cancer was not validated^31^. Recently GRAIL has reported on their pan-cancer assay that relies on a hybrid capture and whole-genome bisulfite sequencing (WGBS) approach and has also shown promise, though the sensitivity for all stages of breast cancer was 33.2% at 99.4% specificity^32^. A similar but more targeted hybrid capture and NGS sequencing approach also provided good detection and tissue of origin identification in advanced cancers^69^. These approaches are intended as pan-cancer screening tests but require levels of sequencing 100-fold or greater that a targeted approach such as mDETECT.

mDETECT_TNBC_ was shown to offer better detection and quantification of tumour burden compared to CA15.3, which is currently the main marker used in the metastatic setting. This assay would thus be more useful for the monitoring of disease burden in the metastatic settings where it could be used to guide decisions around chemotherapy use. We expect it would also have use in locally advanced disease where disease burdens are greater. With *BRCA1* carriers being at high risk of developing this form of breast cancer, mDETECT_TNBC_ may also play a role in the early detection of disease in this population.

## Methods

### Probe development

Level 3 Illumina 450 K methylarray data from The Cancer Genome Atlas (TCGA) (https://cancergenome.nih.gov/) was obtained for tumours of the TNBC subtype based on the basal PAM50 molecular signature as well as ER, PR and HER2-negative status and data from tumours with *BRCA1* mutations were also analysed. This data was also obtained for normal breast, lung, colorectal and prostate samples and averaged. CpG residues that were differentially methylated between tumours and normal tissues were identified where at least 50% of tumours had a methylation value greater than −0.1 and where each of the normal tissue averages was less than −0.3. CpGs were identified only if two probes meeting the outlined characteristics were within 300 base pairs of each other. Seven additional regions were developed from regions found to be prognostic indicators for TNBC^27^ and a probe for methylation of the *BRCA1* promoter^40^ was also developed.

Each of the identified regions were characterised by examining the flanking CpGs around the initially identified region for concerted differences in methylation and a target defined. These were further characterised to ensure that they were not methylated in normal blood cells using the UCSC Genome Browser^70^ and MethBase data tracks^36^. The sequence of these regions were altered to the sequence of fully methylated bisulphite converted DNA using Methyl Primer Express Software v1.0 (ThermoFisher Scientific). PCR primers to these regions were developed using either Methyl Primer Express Software v1.0 or Primer Blast (National Center for Biotechnology Information, Bethesda, MD, USA)^71^ with the following settings: minimum PCR product size 75 bp, maximum 150 bp; melting temperature minimum 64 ^o^C, optimal 68^ o^C, maximum 72 ^o^C, maximal differential 5 ^o^C, primer size minimum 21 bp, optimal 28 bp, maximum 31 bp. Primers with at least two CpG residues in the region between the primers were chosen. CS1 and CS2 universal primer sequences were added to the 5’ ends of the primers.

One-hundred thirty-six additional regions had previously been developed using the same parameters for the other subtypes of breast cancer (Luminal A, Luminal B, HER2 + and normal-like) (results not shown). These probes were examined in the basal and *BRCA1*-associated tumours in the TCGA data and any positive in over half of either population were also included in the assay.

### DNA isolation and preparation

DNA was isolated from cell lines using the GenElute Mammalian Genomic DNA Mini-Prep Kit (Sigma-Aldrich). DNA from the TNBC tumours used in this study were previously described^72^ as was the procedure for extracting patient and control plasma DNA^44^. DNA was extracted from plasma or serum using the QiaSymphony system (Qiagen), with the QIAmp circulating nucleic acid kit (Qiagen). All samples were bisulfite converted using the EpiTect Fast DNA Bisulfite Kit (Qiagen).

### Characterisation of probes in cell lines and tumours

DNA from six breast cell lines (184-hTERT, MCF-10A, MCF7, SKBR3, MDA-MB-231, HCC1937 and MDA-MB-436), as well as buffy coat DNA from a healthy volunteer and 14 TNBC tumours from the Ontario Tumour Bank (OTB) were analysed. Singleplex PCR was carried out for all cell lines, as well as the buffy coat DNA using Hot Star Taq (Qiagen) with 35 cycles of PCR followed by barcoding. Multiplex PCR using Qiagen Multiplex PCR Plus mix was carried out to assess the 14 OTB TNBC tumours.

### Multi-Singleplex assay on plasma and serum

The volumes of the samples ranged from 0.5 ml to 1.8 mls and in some cases had not been subjected to high speed centrifugation. DNA was extracted from plasma and serum samples using the QIAamp Circulating Nucleic Acid kit (cat. 55114) following the manufacturer’s protocol and was eluted in 60 µl. Bisulfite conversion was conducted using the Qiagen EpiTect® Fast Bisulfite Kit (Cat. No. 59824) using 40 µl of the extracted DNA and the converted DNA was eluted in 25 µl of buffer with 10 µl being used in the subsequent Multiplex PCR (a total of 25% of the original sample). A linear multiplex mix of all negative strand primers (reverse) was first carried out to avoid dilution of the ctDNA in subsequent PCR reactions. The linear PCR was completed with the following reagents in a total volume of 25 µl: 1x Multiplex PCR Plus buffer (Qiagen), 0.02 µM each reverse primer, and 10 µl bisulfite converted serum or plasma DNA. PCR conditions were 95 °C (15 min), 50 cycles of 95 °C (30 s), 62 °C (90 s), and 72 °C (30 s), followed by 60 °C (5 min). The products were purified using AMPure XP beads at a ratio of 1:1.2 (sample:beads) and eluted in 50 µl. Individual primary PCR reactions were carried out with each primer pair in a total volume of 10 µl: 1x Hot Star Taq buffer (Qiagen), 0.2 mM dNTPs, 0.2 units Hot Star Taq, 1.5 mM MgCl_2_, 10 µl primary PCR product, and 0.2 µM each primer. PCR conditions were 95 °C (15 min), 35 cycles of 95 °C (30 s), 62 °C (30 s), and 72 °C (30 s), followed by 72°C (5 min). Following amplification 2 µl of each product was pooled and purified using AMPure XP beads at a ratio of 1:1.2 (sample:beads) and eluted in 25 µl of water. Secondary barcoding PCR was used to add Ion Torrent sequencing primers and barcodes for each sample. Total volume of 50 µl: 1x Hot Star Taq buffer (Qiagen), 0.2 mM dNTPs, 1 unit Hot Star Taq, 3 mM MgCl_2_, 10 µl primary PCR product, and 0.08 µM each CS1 barcode P7 adapter and CS2 P1 adapter. PCR conditions were 95 °C (15 min), five cycles of 95 °C (30 s), 58 °C (30 s), and 72 °C (30 s), followed by 60 °C (5 min). Each product was pooled and purified using AMPure XP beads at a ratio of 1:1.2 (sample:beads) and eluted in 30 µl of water, then quantified. Samples were sequenced using the Ion Torrent PGM or S5 machines. Sequencing-generated BAM files for each sample were uploaded to the Galaxy web platform^73^ for processing prior to methylation analysis. The bam sequence files were converted to FASTQ format (SAM to FASTQ ver 1.56.1),filtered to retain reads >100 bp (Filter Fastq ver 1.0.0), converted to FASTA format (FASTQ to FASTA ver 1.0.0), and separated by using the bisulfite sequencing primer sequences (demultiplexing) (Barcode Splitter ver 1.0.0). FASTA sequencing files were mapped to reference sequences and analysed for methylation using BiQ Analyser HT (ver 3) and HiMod (ver 5.1)^74,75^.

### Clonal hematopoiesis of indeterminate potential

Clonal hematopoiesis of indeterminate potential (CHIP) was identified in peripheral blood samples according to previous published experience using an amplicon panel covering 48 CHIP/myeloid cancer-related target genes^76^. Briefly, barcoded libraries were prepared from 15 ng of extracted buffy coat DNA and templated for sequencing using the Ion Chef system, then sequenced using the Ion S5XL System. Sequences were aligned to the human genome hg19, variants called in Ion Torrent Suite (ver 5.14) and annotated in Ion Reporter. Variants (SNVs and indels) were filtered based on the following inclusion rules: exonic location, non-synonymous substitution, *p*-value < 0.01, coverage > 50, VAF between 0.02 and 0.43. Furthermore, variants were excluded if: present in UCSC Common SNP Database, minor allele frequency (MAF) > 0.02, Global/European/American MAF > 0.02. This filtered list was visually inspected in the Integrative Genomics Viewer (ver2.9) (IGV, Broad Institute) to rule out false positives (on amplicon edges, strand bias, poor mapping quality, and presence in other samples.

### Ethical approval and consent to participate

Plasma was obtained from the previously characterised Cohort 03 and were patients treated at the Institut Curie (Paris, France) for metastatic TNBC starting a new line of therapy^44^. Written informed consent was obtained and the study approved by regional ethics boards. Patients in the CRB cohort gave their informed consent to participate in the “ALCINA” blood biomarkers study (NCT02866149). Control serum and plasma samples were obtained from the community with ethics approval from the Health Sciences Research Ethics Board of Queen’s University, Canada, reference number DBMS-052-15.

### Reporting summary

Further information on research design is available in the Nature Research Reporting Summary linked to this article.

## Supplementary information

Supplementary Information

Reporting Summary

## Data Availability

The data that support the findings of this study are available from at NCBI SRA BioProject accession PRJNA733510.

## Code availability

All software was publically available and version numbers are indicated in the methods.
