# Supplementary material for: A DNA methylation-based liquid biopsy for triple-negative breast cancer
Source: NPJ Precis Oncol. 2021 Jun 16;5:53. doi: 10.1038/s41698-021-00198-9 (PMC8209161; doi:10.1038/s41698-021-00198-9)
Supplement: Supplementary file 1 — Supplementary Information [file 41698_2021_198_MOESM1_ESM.pdf]

Supplementary Table 1 List of primers making up the mDETECT$_{TNBC}$ assay and their positivity based on TCGA data.

| Gene | Primer name | 5' - 3' Primer Sequence (Bisulfite) | Chr Location (hg18) | Length | CpGs Primer | Product size | CpG Target | TCGA Probe | Location (hg18) | Basal | Her2 | LumA | LumB |
|---|---|---|---|---|---|---|---|---|---|---|---|---|---|
| BRCA1[1] | BRCA1 Af | GGTAACGGAAAAGCGCGGGAATTATAGA | chr17:38530874-38530968 | 28 | 3 | 95 | 5 | chr17 | 38530954 | 0.09 | 0.00 | 0.00 | 0.00 |
| | BRCA1 Ar | CCCACAACCTATCCCCCGTCCAAAAA | | 26 | 1 | | | | | | | | |
| Intergenic 5 | Int5f | TAGTGATTGGTTATTTGGGCGCGGGGC | chr10:43138416-43138530 | 27 | 2 | 115 | 5 | chr10 | 43138435 | 0.51 | 0.81 | 0.52 | 0.78 |
| | Int5r | AAACGACATCCATCATCTCCCTCGACCC | | 28 | 2 | | | | | | | | |
| ACVRL1 | ACVRL1f | GGATGTGGGAGGTTCGGTTCGGGTG | chr12:50587308-50587443 | 25 | 2 | 136 | 10 | chr12 | 50587574 | 0.38 | 0.90 | 0.70 | 0.87 |
| | ACVRL1r | CCGCTCGCCCCTCGCTAAAACTACA | | 25 | 3 | | | | | | | | |
| BARHL2 | mbBARHL f | TGGTTTTTTTCGGTTTTTGTTCGA | chr1: 90967815 - 90967900 | 24 | 2 | 86 | 4 | chr1 | 90967924 | 0.53 | 0.94 | 0.76 | 0.87 |
| | mbBARHL r | ACTTTCTCCCAATTCCAATATCCA | | 24 | 0 | | | | | | | | |
| CA9 | CA9 Af | TTTCGGGCGGGAGTATCGGGTTTTGTAG | chr9:35666101-35666239 | 28 | 3 | 139 | 6 | chr9 | 35666166 | 0.69 | 0.74 | 0.32 | 0.57 |
| | CA9 Ar | GCTCCTTTACCCCTTCTCGACCAACTCC | | 28 | 1 | | | | | | | | |
| CARD11 | CARD11 Bf | TTTAGGGGTTGTAGGGTCGGG | chr7:3049955-3050087 | 21 | 1 | 133 | 5 | chr7 | 3050067 | 0.70 | 0.77 | 0.55 | 0.65 |
| | CARD11Br | ATTTTACATTTCCCTCCCCCGC | | 22 | 1 | | | | | | | | |
| CCL28 | CCL28 Af | AGGGTGGAGTTTTAGGTAGCGC | chr5:43433001-43433128 | 22 | 1 | 128 | 16 | chr5 | 43433045 | 0.68 | 0.23 | 0.17 | 0.20 |
| | CCL28 Ar | AACAACCCGCGATAAACTAAACC | | 23 | 2 | | | | | | | | |
| CD38[2] | CD38f | GCGATTAAGGCGTATCGGTGGGTATTGC | chr4:15389377-15389501 | 28 | 3 | 125 | 10 | chr4 | 15389336 | 0.20 | 0.77 | 0.65 | 0.79 |
| | CD38r | AACACCACCCGACGAACTCTCGACTAAC | | 28 | 3 | | | | | | | | |
| CDKL2 | CDKL2 Af | ATTGGTCGAGTCGAGTCGTTAC | chr4:76774785-76774935 | 22 | 3 | 151 | 5 | chr4 | 76774837 | 0.62 | 0.65 | 0.35 | 0.46 |
| | CDKL2 Ar | ACAAAAAAACGCCTCCTAACGAA | | 23 | 2 | | | | | | | | |
| CHST11[2] | CHSAf | GGGCGGCGTGGGAATGAATTTT | chr12: 103376278 - 103376397 | 22 | 2 | 120 | 6 | chr12 | 103376485 | 0.07 | 0.42 | 0.49 | 0.65 |
| | CHSAr | CTTTCCCTCGCACCCCTAAA | | 20 | 1 | | | | | | | | |
| DMRTA2 | pbDMRTA f | CGAAGATTTCGTAGGCGGGT | chr1: 50659325 - 50659469 | 20 | 3 | 145 | 12 | chr1 | 50659370 | 0.68 | 0.94 | 0.42 | 0.64 |
| | pbDMRTA r | ACGACGCAAATAACGCTACGCA | | 22 | 4 | | | | | | | | |
| DMRTA2 | DMRTAexp Af | GCGGCGGTTAGCGTTAGTTTTTCGGTAG | chr1: 50659366 - 50659489 | 28 | 4 | 124 | 8 | chr1 | 50659508 | 0.67 | 0.87 | 0.66 | 0.86 |
| | DMRTAexp Ar | CGAAACGCCAACGTATCATAACGACGCA | | 28 | 5 | | | | | | | | |
| DPP10 | DPP10 Af | CGGATTGCGGGAAGAAGGTAC | chr2:115635638-115635739 | 21 | 2 | 102 | 9 | chr2 | 115635479 | 0.33 | 0.32 | 0.20 | 0.33 |
| | DPP10 Ar | GACGAAACGAAACCAAACGACAA | | 23 | 3 | | | | | | | | |
| DPP10 | DPP10 Bf | CGGTTTTCGAGTTTGAAGCGTTC | chr2:115635947-115636088 | 23 | 3 | 142 | 16 | chr2 | 115636255 | 0.32 | 0.35 | 0.35 | 0.48 |
| | DPP10 Br | TACCGACTCTCACCTAATCCGC | | 22 | 2 | | | | | | | | |
| DRD4 | DRD4 Af | CGTCGGGCGATGTTGGTTTGTTCGTG | chr11:627035-627175 | 26 | 4 | 141 | 8 | chr11 | 627038 | 0.56 | 0.68 | 0.39 | 0.61 |
| | DRD4 Ar | GCGACGCTCCACCGTAAACCCAATATTTA | | 29 | 3 | | | | | | | | |
| EFNA4 | ERNA Bf | TGTGCGAAAGAGACGCGGGGTTTAGTTA | chr1:153310139-153310288 | 28 | 3 | 150 | 9 | chr1 | 153310037 | 0.56 | 0.90 | 0.60 | 0.78 |
| | ERNA Br | CCCGTAATCGCTAAAACATCCGCCCTTA | | 28 | 3 | | | | | | | | |
| EVX1[2] | EVX1f | AGGAGGATGATAGTTTAGAAAGAAGAGGGT | chr7:27248900-27249019 | 30 | 0 | 120 | 4 | chr7 | 27248956 | 0.45 | 0.84 | 0.57 | 0.81 |
| | EVX1r | CGCGACCGCGACGATAACGATAAAAACT | | 28 | 6 | | | | | | | | |
| GNG4 | mbGNG f | GTAGGTTTTTGCGTTGGAGATT | chr1: 233880677 - 233880817 | 22 | 1 | 141 | 4 | chr1 | 233880662 | 0.36 | 0.90 | 0.78 | 0.89 |
| | mbGNG r | ATTTTCGTTACTTCTCTATTCCCAAA | | 26 | 1 | | | | | | | | |

| | | | | | | | | | | | | | |
|---|---|---|---|---|---|---|---|---|---|---|---|---|---|
| HNF1B[2] | HINF Cf | GTTAAGGTTTAGTTTCGAAATCGC | chr17: 33177654 - 33177773 | 24 | 2 | 120 | 8 | chr17 | 33178066 | 0.38 | 0.48 | 0.20 | 0.43 |
| | HINF Cr | CTAAAAAACCGAACGATTCCCTAA | | 24 | 2 | | | | | | | | |
| HOXA9 | HOXAAf | GTAATAATTTGGTGGTATCGGGGG | chr7: 27171666 - 27171765 | 24 | 1 | 100 | 5 | chr7 | 27171755 | 0.51 | 0.52 | 0.59 | 0.75 |
| | HOXAAr | TCTACTAAACGAACACGTAACGC | | 23 | 3 | | | | | | | | |
| HOXA9 | HOXACf | TGGGGTTTGTTTTAATTGTGGTT | chr7: 27171878 - 27172029 | 23 | 0 | 152 | 5 | chr7 | 27172029 | 0.54 | 0.55 | 0.46 | 0.62 |
| | HOXACr | GCGAAACCCGCGCCTTCTTAAT | | 22 | 3 | | | | | | | | |
| HOXB13[1] | HOXB13 Af | TCGCGGGTTATAAATATTTGGTTGCGGC | chr17:44157793-44157885 | 28 | 3 | 93 | 4 | chr17 | 44157887 | 0.59 | 0.90 | 0.31 | 0.51 |
| | HOXB13 Ar | GACCGCCACTACCTCGAAAACATTTCCC | | 28 | 2 | | | | | | | | |
| ID4[2] | mbID f | GGAATTTTACGGATGGAGTGATG | chr6: 19945031 - 19945148 | 23 | 1 | 118 | 6 | chr6 | 19945074 | 0.33 | 0.84 | 0.59 | 0.80 |
| | mbID r | CTTATCCCGACTAAACTACTAAAAAA | | 26 | 1 | | | | | | | | |
| IRF8 | IRF8 Af | TTACGATTTTTTTTAAGGTCGCGC | chr16:84490230-84490341 | 24 | 3 | 112 | 12 | chr16 | 84490169 | 0.37 | 0.39 | 0.36 | 0.53 |
| | IRF8 Ar | ACTATACCTACCTACCGCCGTC | | 22 | 2 | | | | | | | | |
| ITPRIPL1[2] | ITPRIPL1f | TTTTGTACGTTGGGTTACGGGGGTTTGG | chr2:96354715-96354857 | 28 | 2 | 143 | 14 | chr2 | 96354744 | 0.53 | 0.84 | 0.28 | 0.57 |
| | ITPRIPL1r | TAAACGCGATAAACCCCTACGACCCCCA | | 28 | 3 | | | | | | | | |
| LEF1 | LEF1 Af | AGAGTTGGGGGCGGTATAGTTAGGGTGT | chr4:109307444-109307547 | 28 | 1 | 104 | 4 | chr4 | 109307487 | 0.61 | 0.32 | 0.10 | 0.13 |
| | LEF1 Ar | TTCAATCCCTACGACCCCAACGCCTAAA | | 28 | 2 | | | | | | | | |
| MAST1 | MAST1 Af | GGAGAAGGTGGTCGGTAAGC | chr19: 12839386 - 12839533 | 20 | 1 | 148 | 6 | chr19 | 12839611 | 0.56 | 0.87 | 0.67 | 0.88 |
| | MAST1 Ar | AAAACGTAATTATAAAAAACACGCC | | 25 | 2 | | | | | | | | |
| NFIC | NFIC Bf | TTTTTCGGTTTGAGTTATCGTGGCGGGA | chr19:3386234-3386379 | 28 | 3 | 146 | 12 | chr19 | 3386350 | 0.70 | 0.52 | 0.23 | 0.35 |
| | NFIC Br | CGAACCGTACTTCCAACCAAACGCAACT | | 28 | 3 | | | | | | | | |
| NR5A2 | NR5A2 Bf | GGTAGGGTTTCGGTTGCGTA | chr1:198278432-198278527 | 20 | 2 | 96 | 6 | chr1 | 198278409 | 0.62 | 0.52 | 0.56 | 0.59 |
| | NR5A2 Br | TATTTCCCGAAAACTCCACATCCA | | 24 | 1 | | | | | | | | |
| OTX2[1] | OTX2 Af | AGGGATTGTATTTCGAGGTGGTCGAGGT | chr14:56331673-56331781 | 28 | 2 | 109 | 4 | chr14 | 56331639 | 0.72 | 0.71 | 0.50 | 0.76 |
| | OTX2 Ar | CCGACAAATCGAAACCTTCGCCCGAAAC | | 28 | 4 | | | | | | | | |
| PAX6 | PAX6 Af | GTTAATTTGGATGTTTCGCGTTTC | chr11:31783206-31783322 | 24 | 2 | 117 | 11 | chr11 | 31783150 | 0.60 | 0.71 | 0.61 | 0.84 |
| | PAX6 Ar | GTTTATCGCTACGACCCGACTAA | | 23 | 3 | | | | | | | | |
| PDX1[1] | PDX1exp Bf | GGAAAAAGGAGGAGGATAAGAAGCGCGG | chr13:27396588-27396685 | 28 | 2 | 98 | 6 | chr13 | 27396544 | 0.44 | 0.23 | 0.16 | 0.29 |
| | PDX1exp Br | CTCGCCGAAAATCACGACGCAATCCTAC | | 28 | 4 | | | | | | | | |
| PHOX2B | PHOX Af | GCGGACGTAGTAATGGATTAAACGGGGA | chr4:41447111-41447255 | 28 | 3 | 145 | 5 | chr4 | 41447209 | 0.59 | 0.48 | 0.27 | 0.28 |
| | PHOX Ar | AAATCCGACTCCCTACACTCCCGACTTT | | 28 | 2 | | | | | | | | |
| PPFIA3 | PPFIA3 Af | AGATACGGAGATTTAGCGCGAGATCGGT | chr19:54337953-54338094 | 28 | 4 | 142 | 7 | chr19 | 54337969 | 0.75 | 0.52 | 0.03 | 0.12 |
| | PPFIA3 Ar | AAATTAACCGCCGAACACTCACAATACG | | 28 | 3 | | | | | | | | |
| PRDM13[2] | PRDM13f | AAGTTTCGTCGAGTTGGGGTCGTTGGTT | chr6:100168753-100168844 | 28 | 3 | 92 | 3 | chr6 | 100168693 | 0.05 | 0.52 | 0.23 | 0.39 |
| | PRDM13r | GACCCTTCCCGACAACCATCTCGAACA | | 27 | 2 | | | | | | | | |
| PRKCB | PRKCB Af | TCGGTTCGTAGTTCGCGGTTTC | chr16:23754928-23755052 | 22 | 4 | 125 | 21 | chr16 | 23755057 | 0.30 | 0.68 | 0.71 | 0.83 |
| | PRKCB Ar | GCACGATACTCTCCTCGCCCT | | 21 | 2 | | | | | | | | |
| PRKCB | PRKCB Cf | CGGTAGAAGAACGTGTATGAGGT | chr16:23755076-23755216 | 23 | 2 | 141 | 8 | chr16 | 23755176 | 0.51 | 0.90 | 0.75 | 0.88 |
| | PRKCB Cr | GCTACCCTCGAAAACCCGAA | | 20 | 2 | | | | | | | | |
| PRSS27 | PRSS Af | GGGAGGTTATTCGTAGGATTTGGCGCGG | chr16:2705610-2705748 | 28 | 3 | 139 | 13 | chr16 | 2705711 | 0.78 | 0.39 | 0.15 | 0.20 |
| | PRSS Ar | ATCCTAACGACTACGCACTACTTCCGCA | | 28 | 3 | | | | | | | | |
| PTGDR[2] | TGDRf | AAGAGGGGTGTGATTCGCGAGTTTAGAT | chr14:51804089-51804198 | 28 | 2 | 110 | 5 | chr14 | 51804075 | 0.49 | 0.68 | 0.58 | 0.78 |
| | TGDRr | CCGCGCGCGACTCGAACGAAAAA | | 23 | 6 | | | | | | | | |
| PTPRN2 | PTPRN2 Af | TCGGAGGAGATAAAGGTGTCGC | chr7:157176188-157176342 | 22 | 2 | 155 | 20 | chr7 | 157176209 | 0.71 | 0.61 | 0.51 | 0.63 |

| | | | | | | | | | | | | | |
|---|---|---|---|---|---|---|---|---|---|---|---|---|---|
| | PTPRN2 Ar | CCAACGTACCTAACCCGAAAAC | | 22 | 2 | | | | | | | | |
| SALL3 | SALL3 Af | TGTTTTTTGCGGGTAAGCGTTC | chr18:74841456-74841551 | 22 | 2 | 96 | 12 | chr18 | 74841950 | 0.72 | 0.84 | 0.73 | 0.91 |
| | SALL3 Ar | GCCCACAACTCTCTCGACGAC | | 21 | 2 | | | | | | | | |
| SALL3 | SALL3 Bf | ATTTCGGGAAAGGGTGGGTC | chr18:74840051-74840163 | 20 | 1 | 113 | 11 | chr18 | 74840214 | 0.56 | 0.65 | 0.52 | 0.80 |
| | SALL3 Br | ACCCTAATCCCCCTTCACCA | | 20 | 0 | | | | | | | | |
| SCAND3[2] | pbSCAND f | TTAATTCGTTTCGCGACGTGAG | chr6: 28618249 - 28618359 | 22 | 4 | 111 | 4 | chr6 | 28618292 | 0.54 | 0.65 | 0.78 | 0.83 |
| | pbSCAND r | ACACGCCTTAAAACCTACTCAT | | 22 | 1 | | | | | | | | |
| SIM1[2] | mbSIM Af | AGGCGTTTATTGGTTAATAGGG | chr6: 101019624 - 101019757 | 22 | 1 | 134 | 11 | chr6 | 101019751 | 0.26 | 0.94 | 0.64 | 0.87 |
| | mbSIM Ar | CGACCCGACACCCTAAACTCAT | | 22 | 2 | | | | | | | | |
| SLC7A4 | SLC Af | GAGTTCGTTTAGTTCGTCGGCGTC | chr22:19716858-19717005 | 24 | 4 | 148 | 8 | chr22 | 19716885 | 0.61 | 0.77 | 0.23 | 0.54 |
| | SLC Ar | AACCCCGATAAACTCCGATAACGACCT | | 27 | 3 | | | | | | | | |
| SOX2OT | SOX2OT Bf | GGGTTACGGTTTCGGGTTGT | chr3:182919884-182919969 | 20 | 2 | 86 | 7 | chr3 | 182919839 | 0.53 | 0.77 | 0.60 | 0.80 |
| | SOX2OT Br | CGCGTCGACTCTCAACACTA | | 20 | 3 | | | | | | | | |
| SPAG6 | SPAG6 Af | GTCGAGTCGTCGTTACGATC | chr10: 22674453 - 22674529 | 20 | 4 | 77 | 3 | chr10 | 22674445 | 0.71 | 0.65 | 0.41 | 0.66 |
| | SPAG6 Ar | CTACCCTCCTCGAACTCTACG | | 21 | 2 | | | | | | | | |
| TAL1 | pbTAL f | GTATTGTCGCGGGTTCGTTC | chr1: 47470631 - 47470738 | 20 | 3 | 108 | 4 | chr1 | 47470668 | 0.47 | 0.61 | 0.31 | 0.44 |
| | pbTAL r | CTCAACCAATCCCCACTCCC | | 20 | 0 | | | | | | | | |
| TCTEX1D1 | TCTEX Af | CGGGGAGGGTCGAGGGTTTTGTTTGAG | chr1:66990668-66990782 | 27 | 2 | 116 | 4 | chr1 | 66990723 | 0.71 | 0.71 | 0.43 | 0.65 |
| | TCTEX Ar | GCGTCCCAAACTTCATTCAACCGACGAC | | 28 | 3 | | | | | | | | |
| TMEM132C | TMEM Af | CGGGAGAAAAGTTGTTTCGGTC | chr12:127317663-127317786 | 22 | 2 | 124 | 12 | chr12 | 127317993 | 0.74 | 0.71 | 0.62 | 0.73 |
| | TMEM Ar | CCGCTACGTCTCTACTATCCGA | | 22 | 3 | | | | | | | | |
| TMEM132C | TMEM Bf | GAGTTCGGGGTGAGGGTAGTC | chr12:127318043-127318179 | 21 | 1 | 137 | 11 | chr12 | 127318011 | 0.46 | 0.45 | 0.34 | 0.56 |
| | TMEM Br | GAATCCCGACGCCCAACTAAAAA | | 23 | 2 | | | | | | | | |
| TOB2P1 | TOP Bf | TGATTTGGGTGGATGTAGAGGTTGTGGT | chr6:28283447-28283568 | 28 | 0 | 122 | 7 | chr6 | 28283425 | 0.72 | 0.65 | 0.65 | 0.80 |
| | TOP Br | TTTCGAATAACGCTACTCCGAACCGCGA | | 28 | 5 | | | | | | | | |
| TTBK1 | TTBAf | CGCGGTGTATTGTGGGTAGT | chr6: 43319189 - 43319287 | 20 | 2 | 99 | 10 | chr6 | 43319191 | 0.43 | 0.74 | 0.57 | 0.78 |
| | TTBAr | CCTTCCGACCCGAATCATCC | | 20 | 2 | | | | | | | | |
| UBD | UBD Af | TCGGTTGCGTAAATCGCGTTTTTGGTTG | chr6:29629437-29629564 | 28 | 4 | 128 | 7 | chr6 | 29629547 | 0.59 | 0.45 | 0.46 | 0.68 |

Gene: indicates closest known gene. Chr Location: region amplified by PCR. CpGs Primer: number of CpG residues in each primer. CpG Target: number of CpGs between primers. TCGA Probe Location: location of probe from TCGA dataset that is closest to PCR product. Basal, HER2, LumA, LumB: Fraction of TCGA patients in the indicated subtype positive for each probe. 1 indicates those probes developed for prognostic relevance in basal breast cancer, 2 indicates those probes taken from the set of probes developed for the other subtypes of breast cancer.

## Supplemental Figures

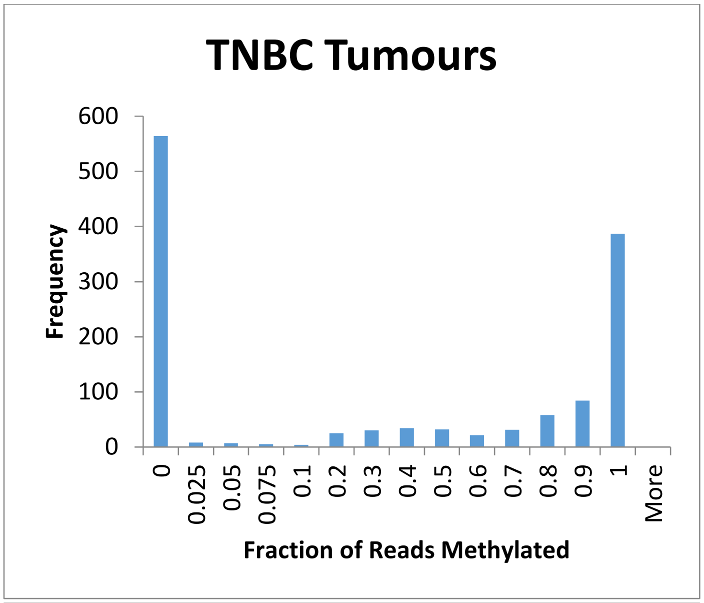


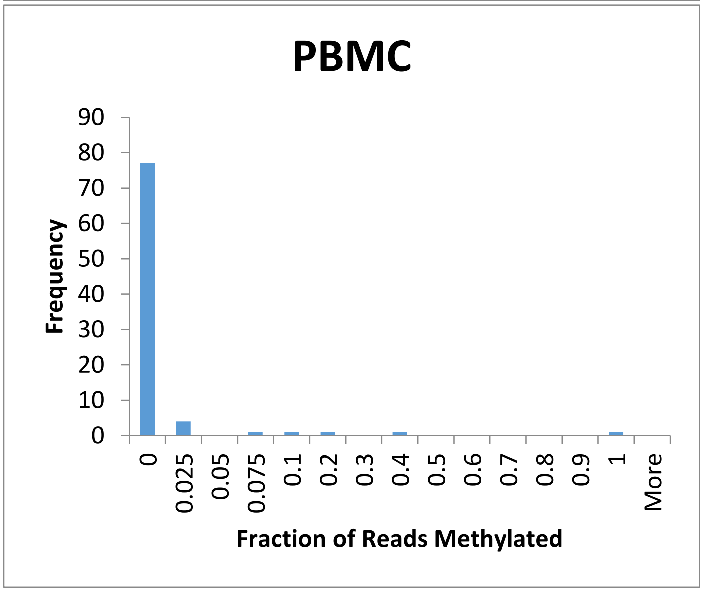


**Supplementary Figure 1: Distribution of Fraction of Reads Methylated in TNBC and Normal PBMC for All Probes.**

For each sample and probe the fraction of reads methylated at all CpG residues between the primers compared to the total number of reads for that probe was calculated. The number of probes in a given range of fractions was then calculated and the frequency per bin is shown for all of the TNBC tumours (TNBC Tumours) or for the normal PBMC DNA sample (PBMC).

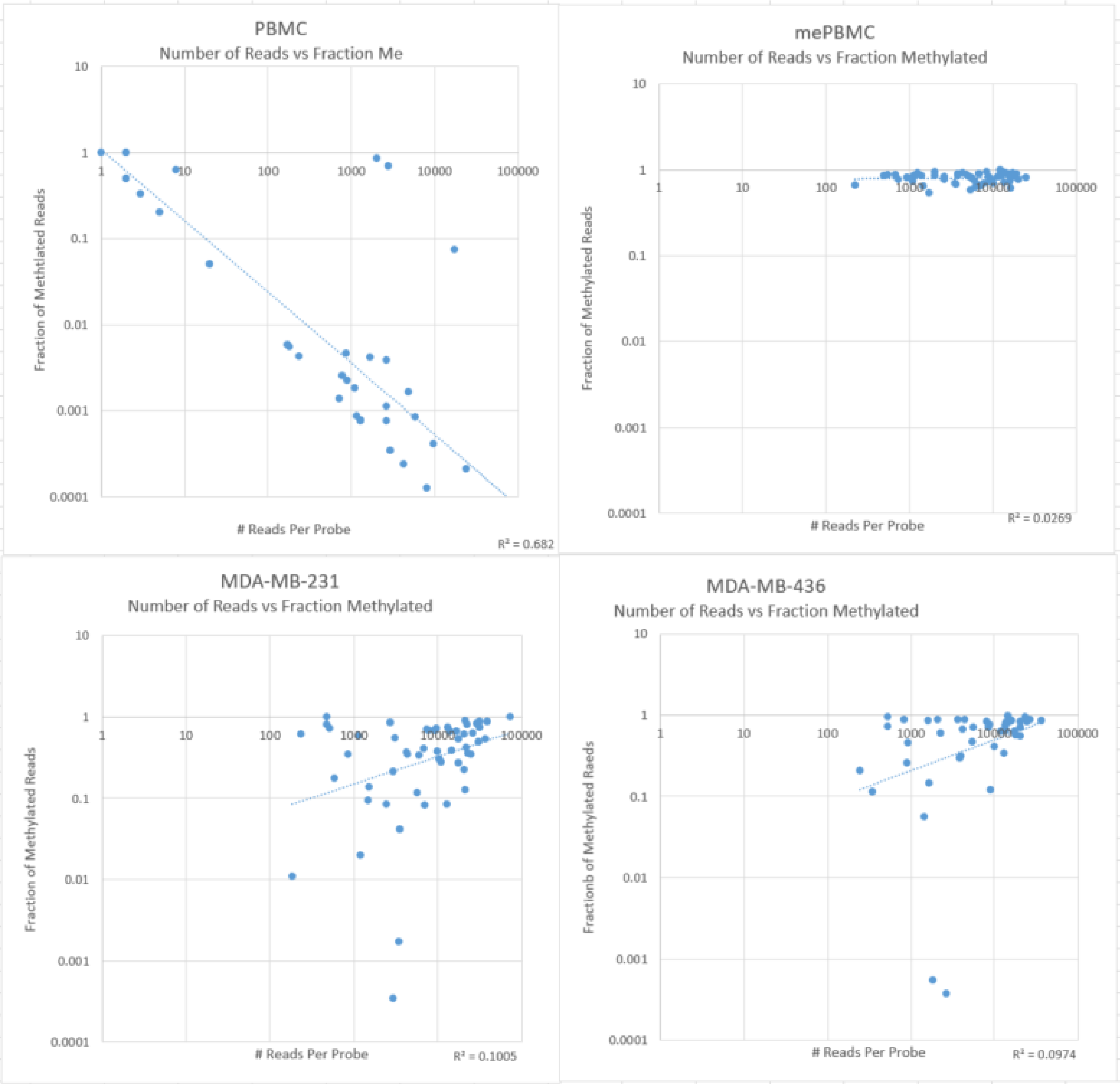


**Supplementary Figure 2: Reads versus Fraction Methylated for each Probe.**

For the Multi-Singlplex protocol the fraction of reads methylated at all CpG residues between the primers compared to the total number of reads for that probe was calculated and is plotted against the total number of reads for that probe. Samples are PBMC DNA from a normal individual (PBMC), the same PBMC DNA synthetically methylated using SSSI methylase (mePBMC), or the TNBC cell lines MDA-MD-231 and 436.

| | | | | mDETECT Value | | | | | | |
|---|---|---|---|---|---|---|---|---|---|---|
| | Days betw | ml Plasma | Age | Discovery | Validation | AVG | ctDNA ng/ | CA 15.3 | CTC | TTP (mont |
| 03-01 | | 1.5 | 37 | | 4 | 4 | 0.798 | 174 | 2 | 22.43333 |
| 03-05 | | 2 | 39 | | 15 | 15 | 44.075 | 35 | 36 | 4.8 |
| 03-06 | | 2 | 75 | | 34 | 34 | NoMut | 24.7 | | -0.3 |
| 03-08 | | 2 | 39 | | 7 | 7 | 0 | 95 | 9 | 0.933333 |
| 03-09 | | 2 | 52 | | 10 | 10 | 0 | 122 | 2 | 4 |
| 03-13 | | 0.5 | 59 | | 30 | 30 | 0 | 266 | 28 | 3.666667 |
| 03-14 | | 0.5 | 50 | | 29 | 29 | NoMut | 28 | | 14.5 |
| 03-15 | | 0.5 | 71 | | 0 | 0 | 3.096 | 29 | 4 | 6.9 |
| 03-16 | | 0.5 | 49 | | 1 | 1 | 0 | 18.5 | 0 | 27.43333 |
| 03-17 | | 0.5 | 59 | 32 | 34 | 33 | 6.457 | 129 | 0 | 5.433333 |
| 03-18 | | | 53 | 30 | | 30 | 6.39 | 84 | 10 | 3 |
| 03-19 | | 0.5 | 57 | 10 | 11 | 10.5 | 0.55 | 24 | 0 | 5.8 |
| 03-20 | | 0.5 | 59 | | 29 | 29 | 4.444 | 36 | 50 | |
| 03-21 | | 0.5 | 57 | | 42 | 42 | 0 | 1839 | 5580 | 7.566667 |
| 03-22 | | 0.5 | 48 | 16 | 12 | 14 | 0 | 21 | 95 | 0.4 |
| 03-23 | | 2 | 47 | 19 | 36 | 27.5 | 10.8 | 26 | 12 | 13.53333 |
| c03-24-1 | 0 | 0.5 | 44 | | 25 | 25 | 40.524 | 106 | 294 | 4.433333 |
| c03-24-2 | 22 | 0.6 | | | 11 | 11 | ND | | | |
| c03-24-3 | 35 | 0.5 | | | 12 | 12 | ND | | | |
| 03-25 | | 1.8 | 48 | 18 | 35 | 26.5 | 6.96 | 30 | | 24.8 |
| 03-26-1 | 0 | 0.5 | 60 | 2 | 2 | 2 | 0.256 | 19 | 0 | 10.2 |
| 03-26-2 | 21 | 0.8 | | | 0 | 0 | ND | | | |
| 03-27 | | 0.5 | 51 | 30 | 34 | 32 | 7.0794 | 103 | 19 | 3.533333 |
| 03-28-1 | 0 | 0.5 | 29 | 15 | 31 | 23 | 2.568 | 33 | 0 | 3.6 |
| 03-28-2 | 62 | 0.5 | | | 14 | 14 | ND | | | |
| 03-29 | | 0.5 | 53 | | 19 | 19 | ND | 260 | | 6 |
| 03-30 | | 1.3 | 64 | 7 | 14 | 10.5 | 10.35 | 90 | 38 | 5.433333 |
| 03-31-1 | 0 | 0.7 | 39 | | 1 | 1 | 0 | 11 | 0 | 1.2 |
| 03-31-2 | 21 | 0.5 | | | 1 | 1 | ND | | | |
| 03-32 | | 0.5 | 57 | | 6 | 6 | 0.3 | 27 | 1 | 0.8 |
| 03-33 | | 0.5 | 52 | | 6 | 6 | 1.106 | 77 | 242 | 1.8 |
| 03-34-1 | 0 | 0.5 | 72 | 9 | 7 | 8 | ND | 25 | | 60 |
| 03-34-2 | 35 | 0.5 | | 2 | 4 | 3 | ND | | | |
| 03-35 | | 0.7 | 46 | | 1 | 1 | NoMut | 15.7 | | |
| 03-38 | | 0.5 | 63 | 43 | 47 | 45 | 107.6 | 94 | 0 | 5.033333 |

**Supplementary Figure 3: Summary of results and characteristics of Validation Cohort.**

Individual patient samples 03-01 to 03-38. Samples with -2 and -3 are from the same patient but at later time points with the time from the first sample indicated in “Days Between”. The volume of plasma extracted for the Validation cohort is indicated (ml Plasma) as is the age of the patient (Age). The mDETECT values for the Discovery or Validation test are indicated as well as the

average of the 2 where applicable. The level of ctDNA determined by TP53 mutation sequencing is shown (ctDNA ng/ml). NoMut refers to no TP53 mutation being found while ND indicates the sample was not done. The levels of CA15.3 and Circulating Tumour Cells per 10 ml of blood (CTC) are indicated. The time to progression (TTP) in months is also shown. Samples that were negative by TP53 mutation detection are indicated in yellow and all were positive for mDETECT.

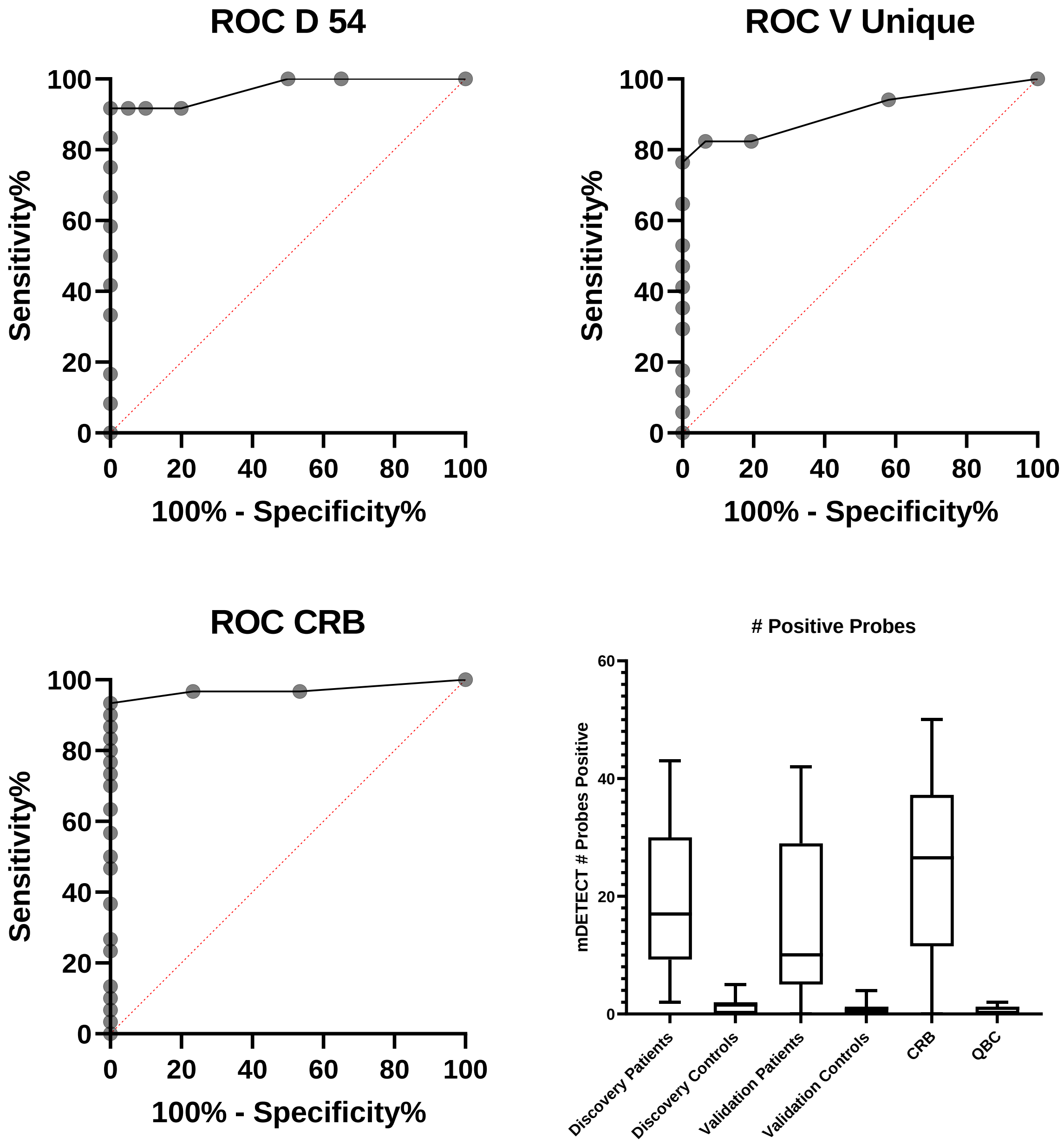


**Supplementary Figure 4: ROC Analysis of the Cohorts.**

The Receive Operator Curves (ROC) for each of the mDETECT analyses of the Discovery Cohort with 54 probes (ROC D 54), the samples from patients unique to the Validation Cohort (ROC V Unique) or from the CRB Cohort are shown (ROC CRB). # Positive Probes. The distribution of number of positive probes for patients and controls is shown for each cohort.

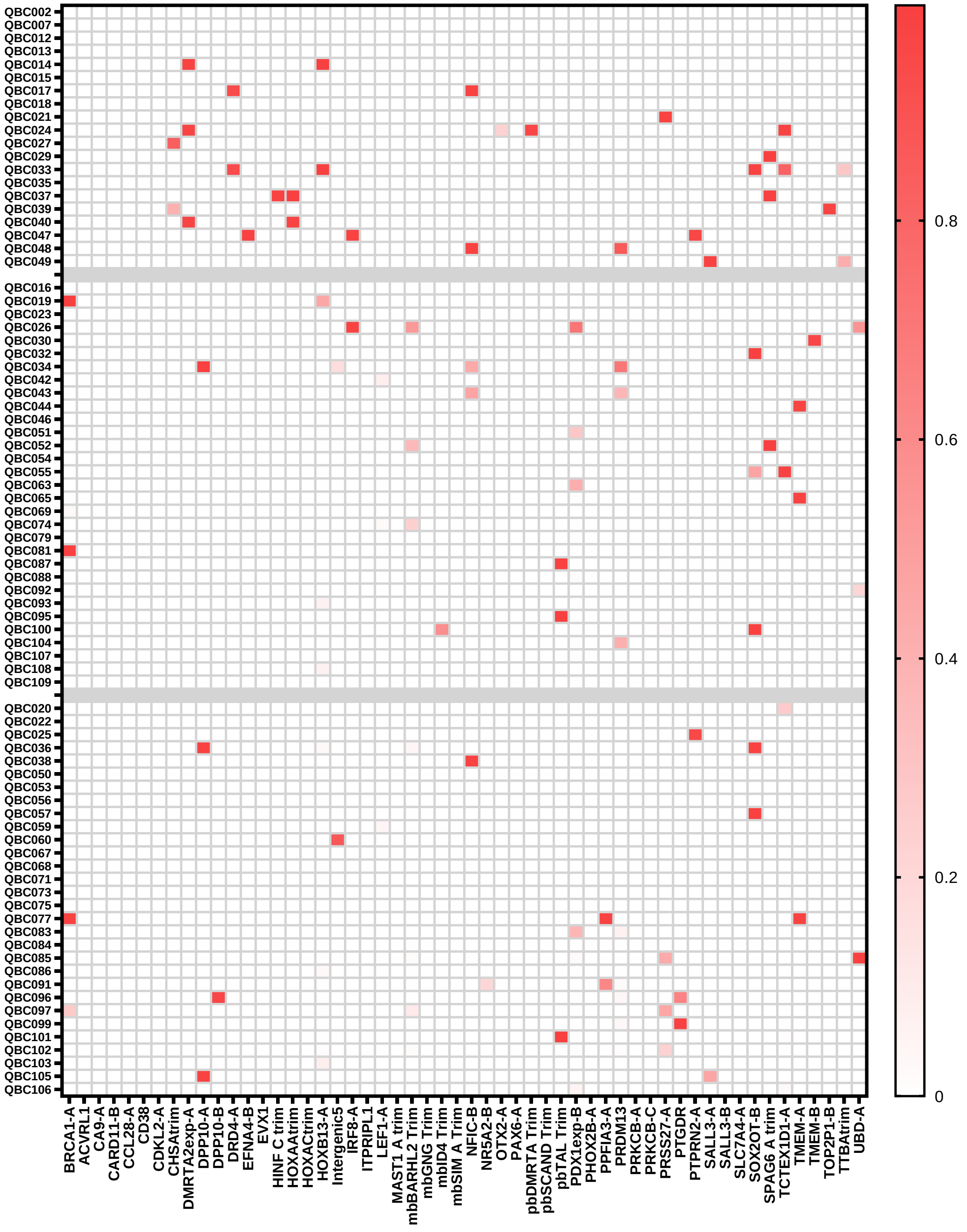

**Supplementary Figure 5: Overall Analysis of QBC Controls.** All of the mDETECT results for the QBC samples are shown with the Discovery Cohort at the top, the Validation Cohort in the middle and the CRB Cohort at the bottom.
