# Supplementary material for: A DNA methylation-based liquid biopsy for triple-negative breast cancer
Source: NPJ Precis Oncol. 2021 Jun 16;5:53. doi: 10.1038/s41698-021-00198-9 (PMC8209161; doi:10.1038/s41698-021-00198-9)
Supplement: Supplementary file 2 — Reporting Summary [file 41698_2021_198_MOESM2_ESM.pdf]

nature research

Corresponding author(s): Christopher Mueller

Last updated by author(s): Apr 12, 2021

Reporting Summary

Nature Research wishes to improve the reproducibility of the work that we publish. This form provides structure for consistency and transparency in reporting. For further information on Nature Research policies, see our Editorial Policies and the Editorial Policy Checklist.

Statistics

For all statistical analyses, confirm that the following items are present in the figure legend, table legend, main text, or Methods section.

n/a	Confirmed	
☐	☒	The exact sample size (n) for each experimental group/condition, given as a discrete number and unit of measurement
☐	☒	A statement on whether measurements were taken from distinct samples or whether the same sample was measured repeatedly
☐	☒	The statistical test(s) used AND whether they are one- or two-sided<br>*Only common tests should be described solely by name; describe more complex techniques in the Methods section.*
☐	☒	A description of all covariates tested
☒	☐	A description of any assumptions or corrections, such as tests of normality and adjustment for multiple comparisons
☒	☐	A full description of the statistical parameters including central tendency (e.g. means) or other basic estimates (e.g. regression coefficient) AND variation (e.g. standard deviation) or associated estimates of uncertainty (e.g. confidence intervals)
☐	☒	For null hypothesis testing, the test statistic (e.g. F, t, r) with confidence intervals, effect sizes, degrees of freedom and P value noted<br>*Give P values as exact values whenever suitable.*
☒	☐	For Bayesian analysis, information on the choice of priors and Markov chain Monte Carlo settings
☒	☐	For hierarchical and complex designs, identification of the appropriate level for tests and full reporting of outcomes
☐	☒	Estimates of effect sizes (e.g. Cohen's d, Pearson's r), indicating how they were calculated

Our web collection on statistics for biologists contains articles on many of the points above.

Software and code

Policy information about availability of computer code

Data collection	These were further characterized to ensure that they were not methylated in normal blood cells using the UCSC Genome Browser(72) and MethBase data tracks(37). The sequence of these regions were altered to the sequence of fully methylated bisulphite converted DNA using Methyl Primer Express Software v1.0 (ThermoFisher Scientific).PCR primers to these regions were developed using either Methyl Primer Express Software v1.0 or Primer Blast (National Center for Biotechnology Information, Bethesda, MD, USA)<br>Sequences were aligned to the human genome hg19, variants called in Ion Torrent Suite and annotated in Ion Reporter. This filtered list was visually inspected in the Integrative Genomics Viewer (IGV, Broad Institute) to rule out false positives (on amplicon edges, strand bias, poor mapping quality, and presence in other samples.<br>Sequencing-generated BAM files for each sample were uploaded to the Galaxy web platform (75) for processing prior to methylation analysis. The sequence files were filtered to retain reads > 100bp, converted to FASTA format, and separated by using the bisulfite sequencing primer sequences (demultiplexing). FASTA sequencing files were mapped to reference sequences and analyzed for methylation using BiQ Analyzer HT and HiMod (76, 77).
Data analysis	Sequencing-generated BAM files for each sample were uploaded to the Galaxy web platform (75) for processing prior to methylation analysis. The sequence files were filtered to retain reads > 100bp, converted to FASTA format, and separated by using the bisulfite sequencing primer sequences (demultiplexing). FASTA sequencing files were mapped to reference sequences and analyzed for methylation using BiQ Analyzer HT and HiMod (76, 77).

For manuscripts utilizing custom algorithms or software that are central to the research but not yet described in published literature, software must be made available to editors and reviewers. We strongly encourage code deposition in a community repository (e.g. GitHub). See the Nature Research guidelines for submitting code & software for further information.

Data

Policy information about availability of data

All manuscripts must include a data availability statement. This statement should provide the following information, where applicable:

- Accession codes, unique identifiers, or web links for publicly available datasets
- A list of figures that have associated raw data
- A description of any restrictions on data availability

The data that support the findings of this study are available from the corresponding author upon reasonable request.

Field-specific reporting

Please select the one below that is the best fit for your research. If you are not sure, read the appropriate sections before making your selection.

☒ Life sciences ☐ Behavioural & social sciences ☐ Ecological, evolutionary & environmental sciences

For a reference copy of the document with all sections, see nature.com/documents/nr-reporting-summary-flat.pdf

Life sciences study design

All studies must disclose on these points even when the disclosure is negative.

Sample size	Samples were limited by the patient cohorts available.
Data exclusions	No data was excluded from these studies
Replication	3 cohorts were use, a discovery cohort, a validation cohort and an additional independent validation cohort using a different source material (serum).
Randomization	Patients were drawn from known cohorts of women with this type of disease, while controls were community members with no known breast cancer
Blinding	Blinding is not relevant as these are women with known disease, or not.

Reporting for specific materials, systems and methods

We require information from authors about some types of materials, experimental systems and methods used in many studies. Here, indicate whether each material, system or method listed is relevant to your study. If you are not sure if a list item applies to your research, read the appropriate section before selecting a response.

Materials & experimental systems

n/a	Involved in the study	
☒	☐	Antibodies
☐	☒	Eukaryotic cell lines
☒	☐	Palaeontology and archaeology
☒	☐	Animals and other organisms
☐	☒	Human research participants
☒	☐	Clinical data
☒	☐	Dual use research of concern

Methods

n/a	Involved in the study	
☒	☐	ChIP-seq
☒	☐	Flow cytometry
☒	☐	MRI-based neuroimaging

Eukaryotic cell lines

Policy information about cell lines

Cell line source(s)	All lines were sources from ATCC
Authentication	not authenticated
Mycoplasma contamination	not tested
Commonly misidentified lines (See ICLAC register)	N/A

Human research participants

Policy information about studies involving human research participants

Population characteristics	Plasma was obtained from the previously characterized Cohort 03 and were patients treated at the Institut Curie (Paris, France) for metastatic TNBC starting a new line of therapy(46). Cohort 03 average age 52.7 (age 29 to 72).<br><br>Control women were an average age of 58.3 with no know breast disease. (age 31 to 71)
Recruitment	Patients were recruited at the Curie Institute by participating physicians<br>Control women were recruited in Kingston Ontario by word of mouth.
Ethics oversight	Written informed consent was obtained and the study approved by regional ethics boards. Patients in the CRB cohort gave their informed consent to participate in the "ALCINA" blood biomarkers study (NCT02866149). Control serum and plasma samples were obtained from the community with ethics approval from the Health Sciences Research Ethics Board of Queen's University, Canada, reference number DBMS-052-15.

Note that full information on the approval of the study protocol must also be provided in the manuscript.
